# Non-motor symptoms in patients with Spinocerebellar ataxia type 12

**DOI:** 10.3389/fneur.2024.1464149

**Published:** 2024-10-14

**Authors:** Purba Basu, Supriyo Choudhury, Siddhartha Sankar Mondal, Ummatul Siddique, Simin Rahman, Jacky Ganguly, Soumava Mukherjee, Nilam Singh, Mona Tiwari, Hrishikesh Kumar

**Affiliations:** Department of Neurology, Institute of Neurosciences Kolkata, Kolkata, India

**Keywords:** SCA12, cerebellum, non-motor features, CAG repeats, cognition, ataxia

## Abstract

**Introduction:**

Spinocerebellar ataxia type 12 (SCA12) is a rare autosomal dominant neurodegenerative disorder caused by abnormal CAG repeat expansion in the *PPP2R2B* gene. This disease is classically characterized by action tremor, dysarthria, ataxia, and hyperreflexia. There are limited reports regarding the non-motor symptoms in patients with SCA 12. We investigated the frequency and factors associated with the selected non-motor symptoms in patients with SCA 12.

**Methods:**

Genetically diagnosed (CAG repeats > 43) and symptomatic cases of SCA 12 were included in the study. Motor severity was assessed using the Scale for the Assessment and Rating of Ataxia (SARA) and The Essential Tremor Rating Assessment Scale (TETRAS). Non-motor symptoms such as depression, autonomic function, and cognition were assessed using the Hamilton Depression Rating Scale (HAM-D), the Scales for Outcomes in Parkinson's Disease-Autonomic Dysfunction (SCOPA-AUT), and the Montreal Cognitive Assessment (MoCA), respectively.

**Results:**

The mean age of the cohort with 34 SCA12 patients was 64.87 years (standard deviation 6.844). In this study, 21 patients (61.76%) had either mild or moderate cognitive impairment, almost equally frequent in early and advanced cases. Similarly a number of patients demonstrated evidence of moderate and severe depression. The SARA score was strongly associated with the MoCA score, and CAG repeat length could independently predict the MoCA score in this cohort. Autonomic symptoms were commonly present, with the majority of the patients experiencing urinary symptoms.

**Discussion:**

Non-motor symptoms are common in SCA12 patients, even in the early stages of the condition. Timely detection and treatment of these symptoms may improve the therapeutic outcome and quality of life for SCA12 patients. The diverse symptoms of SCA12 perhaps indicate a widespread neurodegeneration beyond the cerebellum or its direct connections.

## Introduction

Spinocerebellar ataxia (SCA) type 12 (SCA12) is a rare neurodegenerative disorder with autosomal dominant inheritance. This disease was initially described in the United States of America in a family of German–American descent (1). Subsequently, a majority of affected families have been identified in India, predominantly in the ethnic “Agarwal” community (2). This disease is caused by abnormal expansion of CAG repeat length in the upstream (5′ untranslated) region of the *PPP2R2B* gene. It is classically characterized by action tremor, dysarthria, ataxia, and hyperreflexia (3). There is evidence of extracerebellar involvement in patients with SCA 12. Recent studies have suggested that patients with SCA12 experience substantially reduced cortical excitability, which might be different from other types of Spinocerebellar ataxia (SCAs) (4). In addition, the dopamine reuptake scan was found abnormal in a patient with genetically confirmed SCA12 who presented with atypical Parkinsonism (5). Neuroimaging and neuropathological studies have also supported the evidence of extra-cerebellar degenerative changes in SCA12 patients (6, 7). These preliminary findings indicate that SCA12 may present with symptoms beyond the typical motor symptoms of tremor and ataxic disorder.

There are over 45 types of SCAs with diverse clinical presentations of both motor and non-motor symptoms. Although the existence of non-motor symptoms in SCAs is known, the frequencies are non-uniform across various SCAs (8). There is limited research on the non-motor symptoms of SCA 12. Our group has reported a case series involving 21 SCA12 patients, enlisting their motor and non-motor symptoms (3). In 2021, Agarwal et al. also reported cognitive impairments, especially abnormal executive function and learning disability in SCA12 patients (9), although the factors associated with this cognitive deficit remain unclear. In the current study, we investigated the frequency and extent of the non-motor symptoms and their correlation with the motor symptoms or CAG repeats in a group of patients with SCA 12. In addition, we explored the determinants of the cognitive impairment for this patient population.

## Methods

Genetically confirmed (CAG repeats > 43) and symptomatic patients with SCA 12 were included in the current cross-sectional study. All patients were from the Agarwal community of North Indian origin. Trinucleotide repeat length was obtained by repeat primed polymerase chain reaction (PCR) method. All patients were screened through clinical examinations during their routine visits to the movement disorders clinic of the Institute of Neurosciences, Kolkata, which is a single-specialty neuroscience hospital situated in the eastern part of India. None of the patients had any other pre-existing neurological or psychiatric comorbidities. The study was approved by the Institutional Ethics Committee of the Institute of Neurosciences Kolkata (Ref No. I-NK/MDR/SCA12-Character/2019, dated 19 October 2019), and written informed consent was obtained from the patients before the study-related procedure.

The severity of motor and non-motor symptoms was assessed using validated rating scales. The assessment of the motor symptoms was performed using a standard rating scale for ataxia patients—the Scale for Assessment and Rating of Ataxia (SARA) (10). The disability and impact of the tremor were assessed separately using The Essential Tremor Rating Assessment Scale (TETRAS), given that action tremor is one of the most disabling symptoms of SCA12. The early and advanced SCA12 cases were classified based on TETRAS scores (11). The patients who had either no or slight disability due to the tremor (scores 0/4 and 1/4 of TETRAS) were considered early-stage patients, while those with moderate to severe disability (scores 3/4 and 4/4 of the TETRAS scale) were considered advanced-stage patients. This classification was determined based on a consensus among the authors. The Montreal Cognitive Assessment (MoCA) was used to assess cognitive impairment (12). A score of 18–25 points indicated mild cognitive impairment, 10–17 points indicated moderate cognitive impairment, and <10 points indicated severe cognitive impairment (12). The Hamilton Depression Rating Scale (HAM-D) was used to estimate the symptoms of depression (13–15). Scores below 7 represented absence or remission of depression, scores between 7 and 17 represented mild depression, scores between 18 and 24 represented moderate depression, and scores of 25 and above represented severe depression (16). The quality of sleep/the extent of insomnia was examined using three selected items from the HAM-D. The questionnaire on rapid eye movement (REM) sleep behavior disorder was used for the screening of co-existing REM sleep behavior disorder (17). Autonomic dysfunction was quantified using the Scales for Outcomes in Parkinson's Disease-Autonomic Dysfunction (SCOPA-AUT), where the autonomic control for gastrointestinal, urinary, cardiovascular, thermoregulatory, pupillomotor, and sexual domains was assessed separately (18). The magnetic resonance imaging (MRI) of the brain was performed with a T1-weighted (coronal and axial), T2-weighted, fluid-attenuated inversion recovery, and diffusion-weighted images.

The cerebellar atrophy was estimated by a qualified neuroradiologist (MT), who was blinded to the clinical signs and symptoms of the patient. Atrophy of the vermis and cerebellar hemispheres was visually graded as mild, moderate, and severe, following the criteria reported by Al-Maawali et al. (19). Mild atrophy was identified when few interfoliate sulci were visible, and moderate atrophy was identified when most of the interfoliate sulci were visible with separation of the folia. Cerebellar atrophy was regarded as severe when interfoliate sulci were markedly prominent (19).

The descriptive statistics were presented as mean (standard deviation) for the numerical variables and as percentages for the categorical variables. A more conservative approach was adopted by using non-parametric statistics due to the small sample size included in the current study. The correlation between the two numerical variables was estimated using Spearman's correlation coefficient (rho) and the *p-*value. We also performed modeling through multiple linear regression to identify predictors of non-motor symptoms. In the regression model, the dependent variable was the total MoCA score, and the independent variables were age, disease duration, and CAG repeat length.

## Results

**Figure 1:**
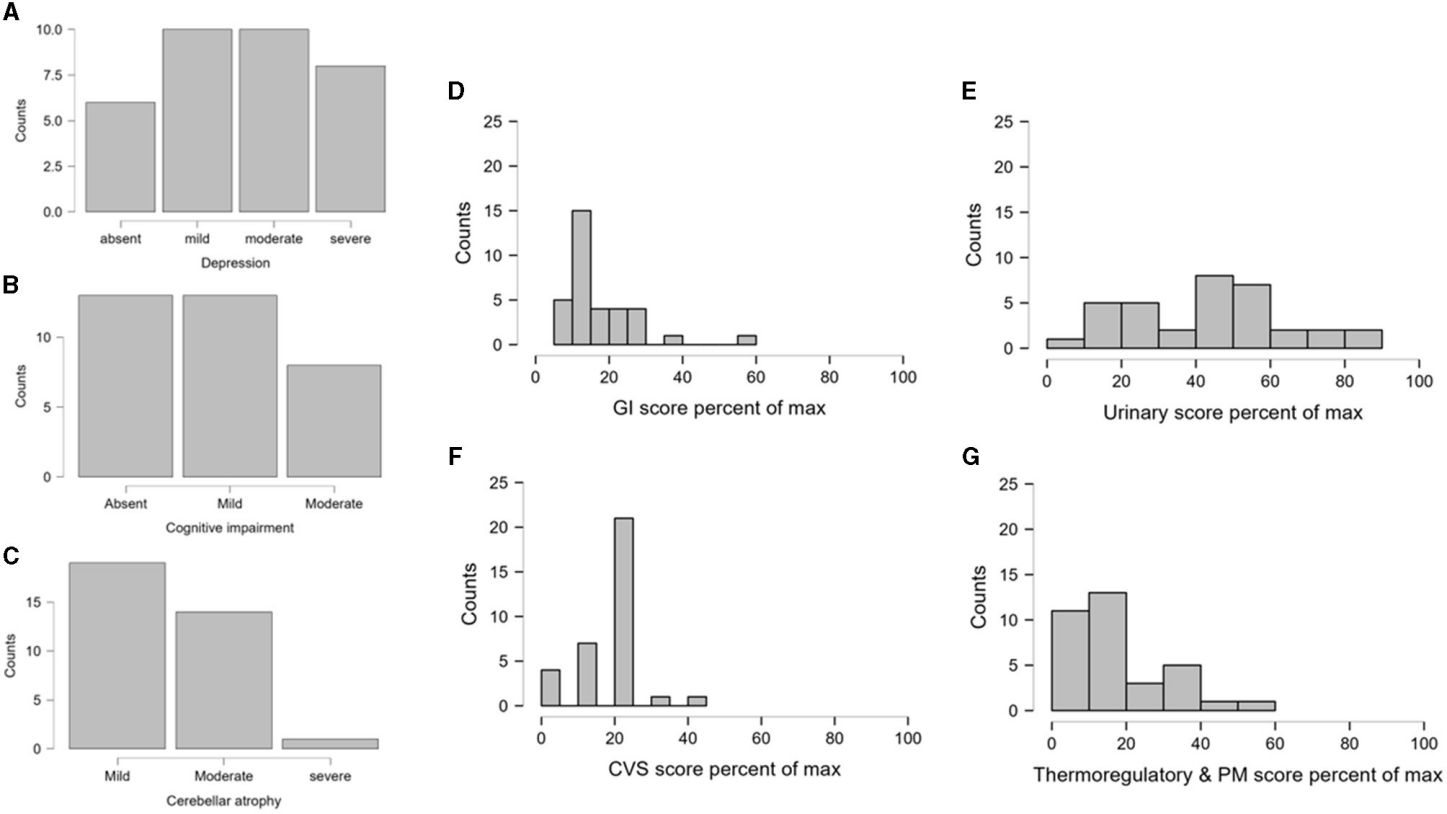
The nonmotor symptoms and cerebellar atrophy of SCA12 patients. **(A–C)** Depicts the frequency distribution of the grades of depression, cognitive impairment, and cerebellar atrophy, respectively. **(D–G)** Is a visual depiction of the four different domains like GI, Urinary, CVS and Thermoregulatory domains of the SCOPA-AUT (estimating autonomic function). The individual scores were expressed as the percentage of the maximum possible scores in the respective domains. The figures present the frequency distributions of the transformed scores as histograms. Sturges' Rule was followed to determine the optimal number of bins in the histograms. GI, Gastrointestinal; CVS, Cardiovascular System; PM, Pupillomotor.

The mean age of the patients was 64.87 ± 6.88 years, and 17 (50%) participants were female patients. The mean CAG repeat length was 52.6 ± 3.7, and the cohort had a mean disease duration of 9.2 ± 3.8 years. All 34 patients had postural and action tremors of varying intensities. The mean TETRAS score was 22.7 ± 10.3, where the maximum possible score on the scale was 42. The overall disability due to tremors was mild or nil in 12 (35.29%) patients. Where as, 22 (64.70%) patients reported significant (moderate to severe) overall disability due to tremors. The mean SARA score for the cohort was 13.8 ± 8.4, where the maximum possible score was 40. Out of the 34 SCA12 patients, 9 (26.47%) patients were either wheelchair-bound or needed strong support to walk. Moreover, 11 (32.35%) patients experienced some degree of staggering while walking. The remaining 14 (41.2%) patients had either a normal gait or a tandem abnormality without staggering. On the visual rating of the MRI scan of the brain, 19 (55.9%) and 14 (41.2%) patients had mild and moderate cerebellar atrophy, respectively. Only one patient had severe atrophy of the cerebellum (Figure 1C).

Figure 1 shows the graphical representation of the occurrence of various non-motor symptoms and grades of cerebellar atrophy.

**Figure 2:**
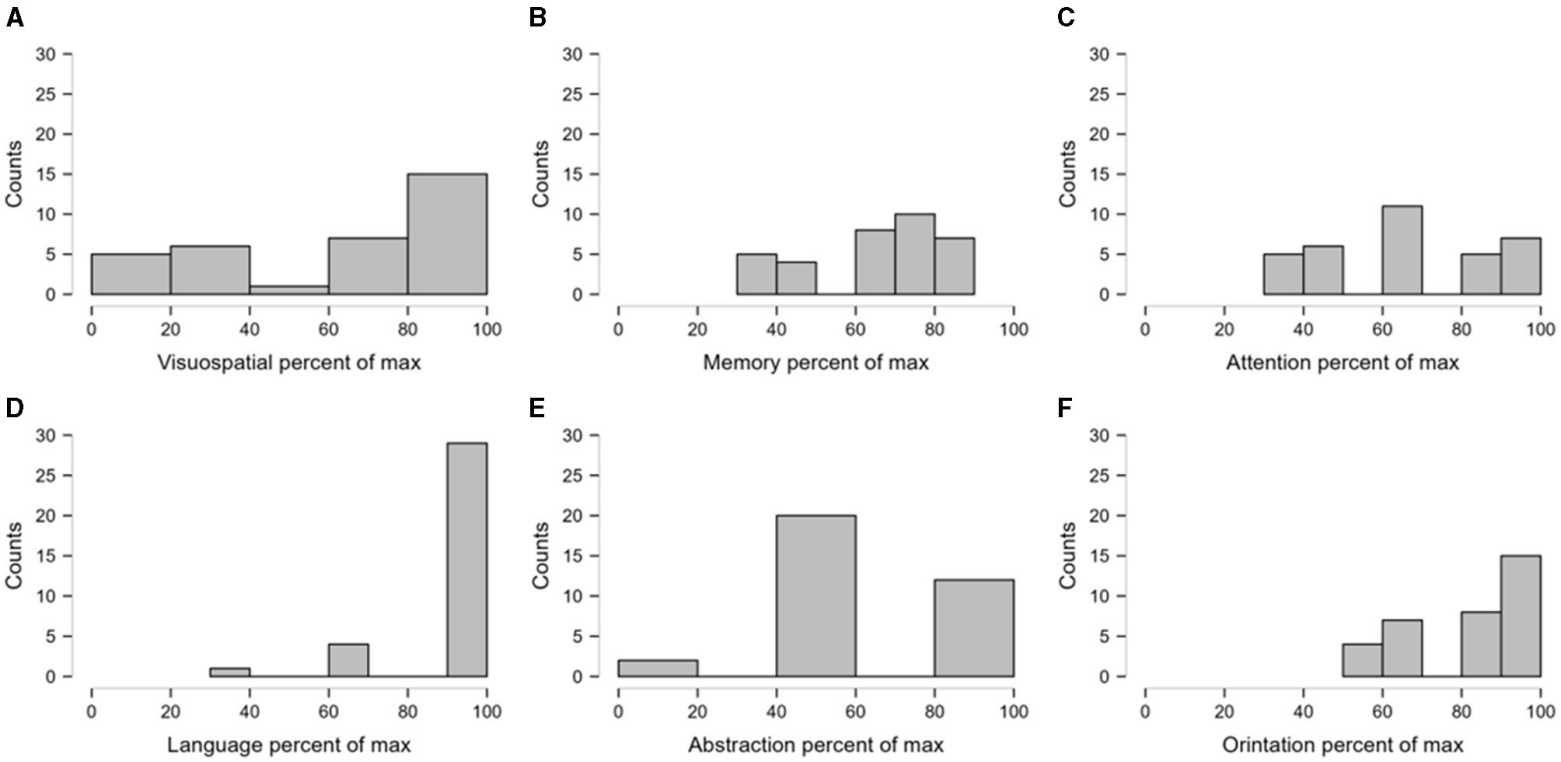
The relative impairment of domains of cognitive score. The individual scores were expressed as the percentage of the maximum possible scores in the respective cognitive domains. The figure presents the frequency distributions of the transformed scores as histograms. Sturges' Rule was followed to determine the optimal number of bins in the histograms. **(A, B)** depict the frequency distribution of percentage of visuospatial and memory where as **(C)** depicts the percentage of attention score. **(D–F)** indicate frequency distribution of percentage of language, abstractation and orientation respectively.

The mean MoCA score was 22.08 ± 5.54% in the cohort of SCA12 patients. Moreover, 13 patients (38.2%) experienced mild cognitive impairment, while 8 (23.5%) patients had moderate cognitive impairment. The individual cognitive domain scores of the MoCA were also expressed as the percentage of the maximum score in the respective domains. In the MoCA, a lower score indicates higher cognitive impairment. Hence, domains with a higher percentage of the maximum score may be considered less affected compared to other domains. In this way, we observed that visuospatial and abstraction were the most affected (<20% of the maximum score in some patients) domains in the current cohort (Figure 2). In addition, 18 (52.94%) patients showed evidence of moderate to severe depression, while 10 (29.41%) patients demonstrated evidence of mild depression, based on the HAM-D cut-off score. Among the early-stage patients, we observed that six (50%) patients had moderate to severe depression (*n* = 12). Similarly, 12 (54.54%) patients reported moderate to severe depression among the advanced patients (*n* = 22).

The SCA12 patients had a median sleep score (selected items from HAM-D) of two [interquartile range (IQR) 1–4], and only one patient was screened positive for RBD. The maximum possible sub-score for sleep was six, as derived from the HAM-D.

Figures 1D–G shows the various domains of autonomic dysfunction. It is apparent that substantial urinary symptoms (>60% of the maximum possible urinary score) were present in patients with SCA12. Autonomic dysfunction leading to Gastrointestinal (GI) and Cardiovascular System (CVS) symptoms was also observed in the group, although less pronounced than the urinary domain.

**Figure 3:**
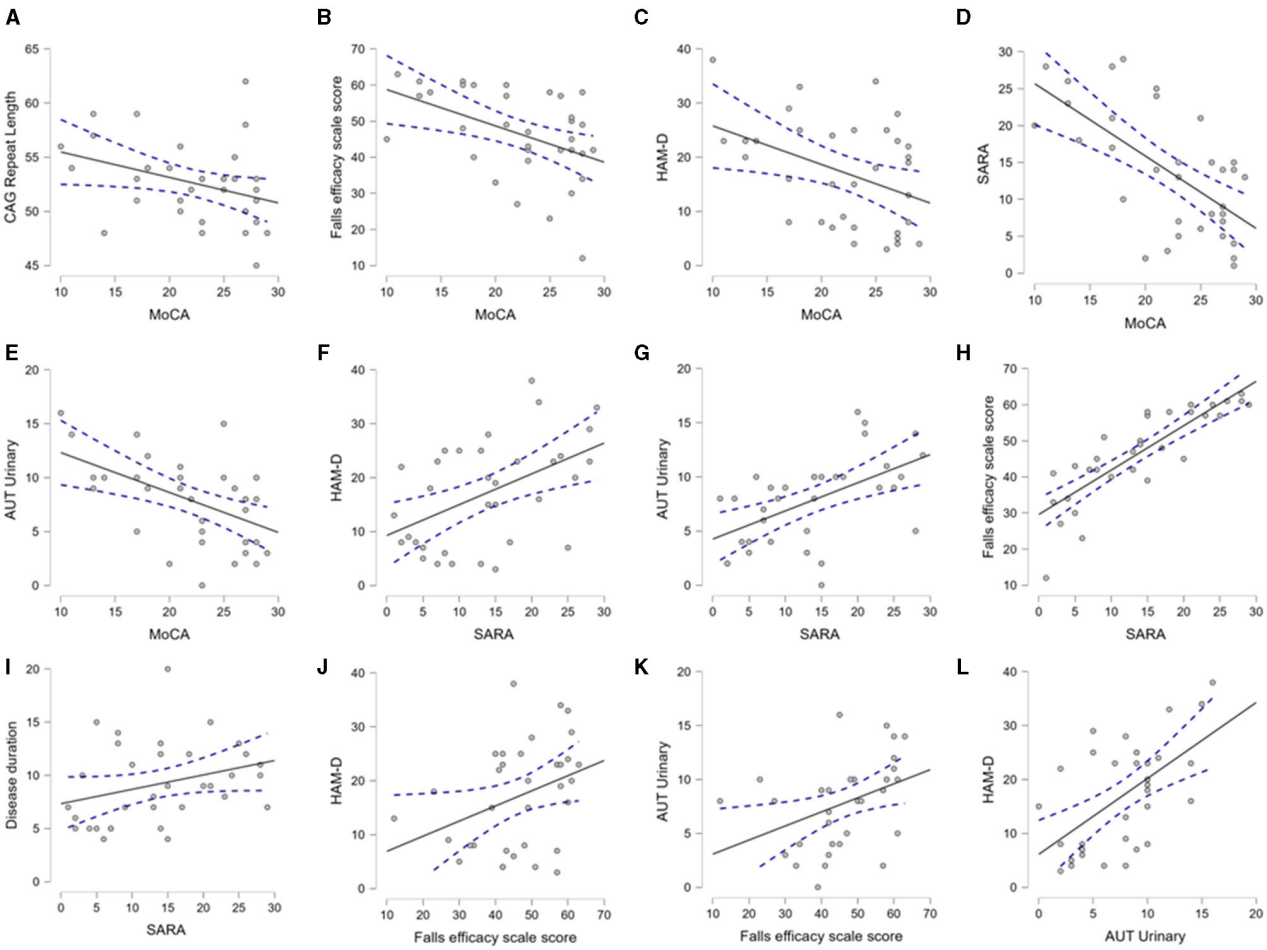
Mutual association between CAG repeat length, demographic and clinical variables. The scatter plots demonstrate the significant association between the clinical, demographic, and CAG repeat length, as analyzed by Spearman's correlation. This figure selectively presents the association between the pairs of variables, where a *p-*value < 0.05 was found. **(A–E)** depict association of MoCA with CAG repeat, Falls efficacy, HAM-D, SARA and Urinary domain of autonomic scores respectively. **(F–I)** demonstrate association of SARA with HAM-D, Urinary, Falls efficacy and Disease duration respectively. **(J, K)** indicate association of Falls efficacy with HAM-D and Urinary domain of autonomic score. **(L)** describes association of autonomic score of urinary symptoms with HAM-D. Aut Urinary: urinary domain of autonomic dysfunction.

After the description of the clinical-demographic and genetic variables, we explored their mutual correlation. In Figure 3, we selectively show the association between the pairs, which were found to have a significant correlation. The MoCA was significantly correlated with the CAG repeat length, Falls Efficacy Scale score, HAM-D score, SARA score, and urinary sub-score of the SCOPA-AUT (Figures 3A–E). The SARA score was correlated with the HAM-D score, Falls Efficacy Scale score, urinary sub-score of the SCOPA-AUT, and disease duration (Figures 3F–I). The Falls Efficacy Scale score demonstrated a correlation with the HAM-D score and urinary sub-score of the SCOPA-AUT (Figures 3J, K). Finally, the urinary sub-score of the SCOPA-AUT was significantly associated with the HAM-D score (Figure 3L). The urinary disturbances were in the form of urgency and urge incontinence. This array of correlations suggests that the factors are interdependent and that one or more factors may predict the cognitive status of SCA12 patients.

We also performed modeling through multiple linear regression to identify predictors of non-motor symptoms. CAG repeat length was found to be an independent predictor of the MoCA score, while adjusted for age and disease duration (R^2^ 0.164, the *p*-value of the CAG repeat length was 0.037). In subsequent models, we observed that the total SARA or TETRAS (motor scores) scores could not be reliably predicted through the mentioned clinical-demographic factors.

## Discussion

In the current study, we presented the non-motor and motor symptoms in a cohort of 34 unrelated SCA12 patients. We observed that moderate to severe depression was present in more than 60% of the patients, and nearly one-fourth of the patients had severe cognitive impairment. The severity of the cognitive impairment was predicted by the CAG repeat length. Severe cerebellar atrophy was identified in only one patient. Autonomic dysfunction was common among SCA12 patients, with urinary symptoms dominating over other autonomic domains. Depression was equally frequent in the early-stage and advanced-stage patients.

In the current study, we observed that 61.8% of SCA12 patients had either mild or moderate cognitive impairment. The most affected domains were visuospatial memory and abstraction.

Cognitive impairment has also been reported in other SCAs. A published study reported that a quarter of SCA2 patients were found to have dementia and early executive dysfunction (20). In another study, cognition was found to be relatively more affected in patients with SCA3 compared to SCA6 patients (21). Frontal and phonemic verbal fluency were found to be altered in a group of SCA10 patients (22). SCA1 patients experienced overall executive dysfunction and mild deficits in verbal memory (23). In a Japanese cohort of patients with SCA3, word retrieval (part of semantic memory), was impaired. In Brazilian and Chinese patients with SCA3, a similar pattern was observed in addition to visuospatial dysfunction in a subgroup (8). SCA7 patients also demonstrated mild to moderate cognitive impairment (24). In a recent report, Indian patients with SCA 12 scored significantly lower than controls in executive function and new learning ability (9). The study also noted that executive function was not correlated with age, age at onset, severity of ataxia, disease duration, and CAG repeat length. In contrast to their findings, we observed that the motor severity was strongly correlated with the cognitive impairment in our cohort. This indicates that progressive neurodegeneration in SCA12 patients is not only restricted to cerebellum and its associated circuit but also affects the cortical and subcortical regions gradually over a period, which may be associated with cognitive control. The lack of correlation between the motor score and executive function score, as reported by Agarwal et al. (9), possibly indicates that the degeneration in SCA12 is not limited to the executive circuit alone but also involves neural substrates related to other cognitive domains (2). Interestingly, CAG repeat length could independently predict the MoCA score in the presence of other possible independent determinants, such as age and disease duration, but the same determinants could not predict the motor (SARA and TETRAS) scores. This independent association between cognitive impairment and causal mutation suggests that the non-motor symptoms are not just an epiphenomenon in SCA12 but a part of the disease phenotype. Nevertheless, the reason for the difference in association between CAG repeat length and cognitive/motor severity is intriguing and needs further studies to elucidate the pathogenesis of these symptoms.

In our study, 52.92% of the patients reported some degree of depression. A similar prevalence of depression (60%) was reported in patients with SCA3 (25). The prevalence of depression in patients with SCA3 and SCA12 seems to be significantly higher than in patients with SCA1, 2, and 6 (23%−27%) (26). Unlike our observation, depression and ataxia were found to be correlated in patients with SCA3. Interestingly, in the current study, we observed that the prevalence of severe depression in the patients with SCA12 was comparable between the early- and late-stage patients. Its prevalence was quite substantial even in the early-stage patients.

In the current study, some degree of autonomic disturbances was present in almost all patients. Autonomic disturbances are frequently reported in other SCAs as well. In one study, more than half of the cohort comprising patients with SCA1, SCA2, and SCA3 was reported to have coexisting autonomic dysfunction, which was most commonly present in patients with SCA3 (27). In another report, parasympathetic abnormality was detected in 70% of these patients (27). Although it was initially considered that SCA6 and SCA12 patients have a relatively preserved autonomic function, a subsequent report demonstrated that half of SCA6 patients were affected by dysautonomia. Another group reported urinary symptoms to be most common in patients with SCA2.

Sleep problems were not a major concern in SCA12 patients, with only one patient tested positive in the RBD screening. Despite previous reports of Parkinsonian symptoms and reduced transporter re-uptake in dopamine imaging (5), RBD was rarely observed in our cohort, unlike in patients with SCA3 where RBD was frequently reported (28, 29).

It is evident that patients with SCA12 present with various non-motor symptoms, which may not be classically explained by the dysfunction of the cerebellum and its associated network. It probably indicates more widespread neurodegeneration across various brain regions. The autopsy study of the brain in one patient with SCA12 revealed generalized cerebral parenchymal atrophy (posterior parietal and bilateral frontal lobes) and atrophy of the pons and cerebellum (7). Neuroimaging studies have also confirmed a reduction in the size of the pons and cerebellum (6). These findings suggest that SCA12 affects multiple brain regions over and above the cerebellum.

The current study has a few strengths and weaknesses.

This is possibly one of the first studies where a large sample size of patients with genetically confirmed SCA12 was carefully examined for motor and non-motor symptoms. An attempt was made to find the correlation among these symptoms.

The cross-sectional nature is one of the limitations of the study. As it was cross-sectional, the order of appearance of the symptoms could not be ascertained. Furthermore, detailed neuropsychological assessments (cognitive function and impulsivity with higher cerebellar involvement, as well as functional impairment due to dementia) and brain morphometry were not performed, which may be marked as a limitation.

Overall, we observed that non-motor symptoms usually co-occur in SCA12 patients. Depression and cognitive impairment are common non-motor symptoms in addition to autonomic dysfunction. The cognitive problem strongly correlates with the greater motor severity of the disease. It could be predicted by higher CAG repeat length in a patient, which suggests that cognitive impairment is part of the disease phenotype and not just a consequential finding. Unlike cognitive impairment, a substantial number of patients were affected with depression even in their early stages. Among cases of dysautonomia, bladder problems are the most prominent, and cardiovascular symptoms are also not uncommon. A careful history and clinical examination for the early detection of non-motor symptoms may improve patient care and therapeutic outcomes in SCA12 patients.

## Data Availability

The original contributions presented in the study are included in the article/supplementary material, further inquiries can be directed to the corresponding author.
